# Functional and Molecular Immune Response of Rainbow Trout (*Oncorhynchus mykiss*) Following Challenge with *Yersinia ruckeri*

**DOI:** 10.3390/ijms23063096

**Published:** 2022-03-13

**Authors:** Carlos Fajardo, Paulo Santos, Ricardo Passos, Mariana Vaz, Rita Azeredo, Marina Machado, Sergio Fernández-Boo, Teresa Baptista, Benjamin Costas

**Affiliations:** 1 Interdisciplinary Centre of Marine and Environmental Research (CIIMAR), University of Porto, 4450-208 Porto, Portugal; carlos.fajardo@uca.es (C.F.); paulo.santos@ciimar.up.pt (P.S.); mleme@ciimar.up.pt (R.A.); mcasimiro@ciimar.up.pt (M.M.); sboo@ciimar.up.pt (S.F.-B.); 2 MARE—Marine and Environmental Sciences Centre, ESTM, Polytechnic Institute of Leiria, 2520-620 Peniche, Portugal; ricardo.passos@ipleiria.pt (R.P.); mariana.c.vaz@ipleiria.pt (M.V.); teresa.baptista@ipleiria.pt (T.B.); 3 Department of Biology, Faculty of Marine and Environmental Sciences, Instituto Universitario de Investigación Marina (INMAR), Campus de Excelencia Internacional del Mar (CEI·MAR), University of Cadiz (UCA), 11510 Puerto Real, Spain; 4 Department of Aquatic Production, School of Biomedicine and Biomedical Sciences, Abel Salazar Institute of Biomedical Sciences (ICBAS), University of Porto, 4050-313 Porto, Portugal

**Keywords:** biomarkers, ERM, hematological profile, oxidative stress, yersiniosis

## Abstract

Currently, aquaculture production of rainbow trout (*Oncorhynchus mykiss*) is a multibillion dollar industry; nevertheless, the development of this sector has not been exempt from pitfalls related to the recurrent presence of pathogens of bacterial origin. This is the case of *Yersinia ruckeri*, the etiologic agent of the infectious pathology known as Enteric Red Mouth Disease (ERM), causing serious economic losses that can be as high as 30–70% of production. Although several studies have been performed regarding pathogen features and virulence factors, more information is needed about the host defense mechanism activation after infection. Given this perspective, this study aimed to evaluate rainbow trout’s short-term innate immune response against infection with *Y. ruckeri*. A series of factors linked to the innate immune response were evaluated, including determination of hematological parameters, oxidative stress biomarkers, and analysis of the expression of immune-related genes. Results showed a significant decrease in several hematological parameters (white blood cell count, hematocrit, neutrophils, monocytes, lymphocytes, and thrombocytes) and oxidative stress indicators (SOD) between the control and infected groups. In addition, there were significant differences in the level of gene expression between infected individuals and the control group. Most of these genes (*il-1β*, *il-8*, *il-10*, *tnf-α1*, *tnf-α2*, *socs3*, *mmp-9*, *cath*, *hsp-70*, *saa*, *fer*, *pcb*) were upregulated within the first 24 h following infection. Results from this study showed more insights into the short-term immune response of rainbow trout to infection with *Y. ruckeri*, which may be useful for the establishment of biomarkers that may be used for the early detection of ERM.

## 1. Introduction

Aquaculture production of rainbow trout (*Oncorhynchus mykiss*, Walbaum 1792) has become a modern multibillion dollar industry, which represents 1.6 % of worldwide aquaculture production (848.1 thousand tons in 2018) [1]. In Portugal, the introduction and cultivation of rainbow trout occurred in the early 1960s, specifically on the banks of the Coura River, and during the last decade, the production has remained relatively stable, oscillating between 900–1000 tons per year [2]. The development of this sector has not been exempt from pitfalls related to the presence of pathogens. Among these, it highlights the recurrent presence of pathogens of bacterial origin, such as the case of *Yersinia ruckeri*, which is a Gram-negative rod with rounded ends. This bacterium does not form spores or capsules, but it presents flagella that provide variable mobility [3], although the existence of non-mobile strains has also been reported [4]. Like the other members of the Enterobacteriaceae family, *Y. ruckeri* is a glucose fermenter, oxidase negative, and nitrate reducer [5]. Biochemical tests of *Y. ruckeri* allow it to be distinguished easily from other *Yersinia* species [6], and PCR is the clinical diagnostic system that has guaranteed the most reliable and specific identification of *Y. ruckeri* in the shortest period of time [7,8].

*Y. ruckeri* was first isolated in the Hagerman Valley (Idaho, EE.USA.) in the early 1950s [5,9]. Currently, *Y. ruckeri* has a worldwide distribution, having been reported in fish populations in America [10,11,12,13], Europe [4,14,15,16,17], Asia [18,19,20], Africa [21], and Oceania [22], causing serious economic losses that can be as high as 30–70% of the stock in some cases [23,24]. *Y. ruckeri* is the causative agent of the infectious pathology known as Enteric Red Mouth Disease (ERM) [9,25,26]. ERM is a systemic infection that can affect trout for almost its entire life cycle [27], with juvenile stages cultivated in freshwater being especially susceptible. Although *Y. ruckeri* can affect different types of salmonid and non-salmonid fish, both in fresh and salt water, *O. mykiss* is the species that has been reported as the most sensitive and vulnerable to ERM [13,28]. ERM has also been referred to as yersiniosis since affected fish do not always present the characteristic reddened areas of the mouth [29,30]. Likewise, this term is used to distinguish a chronic infection from an ERM that appears to be an acute process [31]. As a result of ERM or yersiniosis, exophthalmia with orbital hemorrhages may also occur [9,23,32]. In addition to darkening of the skin and abdominal distension, changes in fish behavior can be observed, such as swimming close to the surface and slow movements. Affected fish are often in a state of torpor and in areas with low water flow, and they often lose their appetite [8].

*Y. ruckeri* infection generally results in the development of acute or chronic septicemia characterized by the presence of hemorrhages around the mouth and anus, at the base of the fins, and on the surface of internal organs [15,32,33,34]. It has been reported that ERM is more contagious when the temperature of the water varies between 15 to 20 °C. The incubation period is about 5 to 10 days [33,35], and disease transmission occurs mainly horizontally through the contact of fish in the water [36,37]. Moreover, once a fish has been infected, it will always carry the pathogen; thus, persistence of *Y. ruckeri* in infected fish and the presence of bacteria in their feces represent a continuous source of infection [9,36]. ERM appears as a pre-acute or acute form of infection, especially in young fish, and is often concomitant with a sudden increase in water temperature during spring. On the other hand, chronic infections generally occur in one-year-old fish, commonly in early winter [15,32,34,37]. Generally, fish have been considered to become infected through external surfaces, such as gills and skin; however, it has also been suggested that the initial target organ of *Y. ruckeri* is the intestine [27,38,39]. Epidermis, dorsal fin, lateral line, and mucous of the gastrointestinal tract are the main areas where invasion by *Y. ruckeri* occurs most easily after exposure. Although some authors do not consider the gills as the primary sites of *Y. ruckeri* invasion [40], others have reported that the challenge through bathing, both *in vitro* and *in vivo*, results in the immediate presence of *Y. ruckeri* in the branchial mucus, which translates into the subsequent invasion of the branchial epithelium and also of the laminar vascular system [41].

Prophylactic strategies against *Y. ruckeri* have been reported for trout since the development of the first commercial fish vaccines, consisting of formalin-inactivated bacteria [24,42]. *Y. ruckeri* can be partly controlled by vaccination; nevertheless, non-epidemic occurrence of ERM may also be seen among farmed rainbow trout [43]. During the last decade, vaccination with bacterins (generally by immersion for 30 s) has been very successful in controlling ERM in cultured rainbow trout [4,44]. Nonetheless, more recently, cases of yersiniosis in cultured rainbow trout were reported in different geographical areas in which systematic vaccination against ERM disease had been performed, such as in England and Spain [4,17]. Given this perspective, and due to the high incidence of ERM in rainbow trout cultured in Portugal, the present study aims to gain new knowledge on the short-term innate host defense mechanisms against *Y. ruckeri* infection.

## 2. Results

### 2.1. Hematological Analyses and Blood Smears

**Figure 1:**
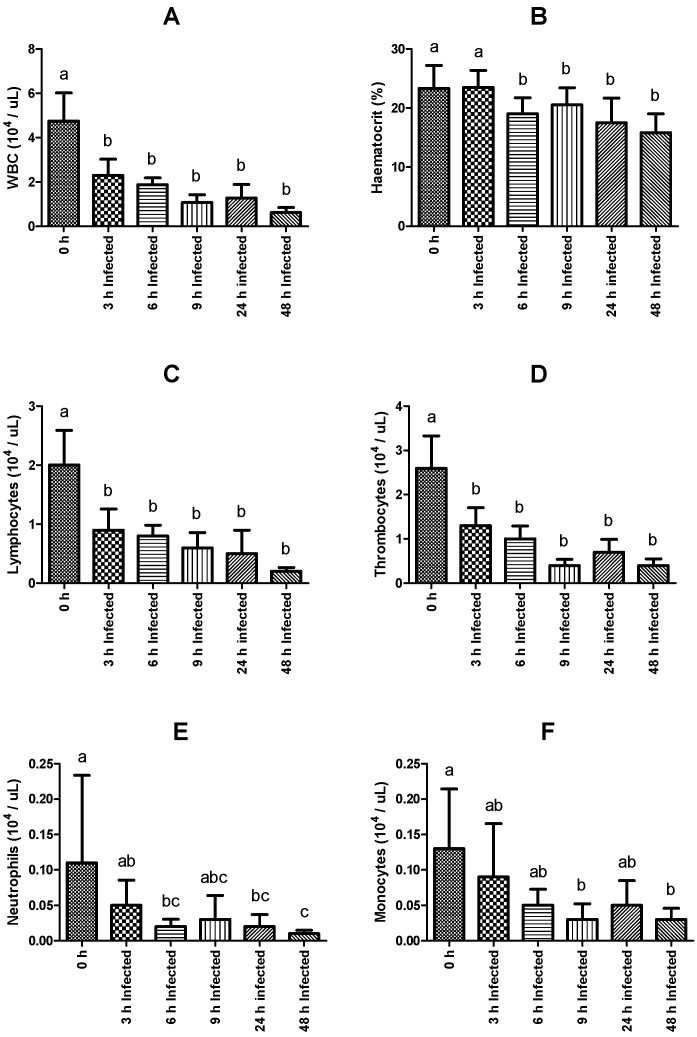
Hematological parameters of rainbow trout sampled before (0 h) or 3, 6, 9, 24, and 48 h post-intraperitoneal infection with *Y. ruckeri* (means ± SD, *n* = 6). (**A**) WBC (white blood cells); (**B**) Hematocrit; (**C**) Lymphocytes; (**D**) Thrombocytes; (**E**) Neutrophils; (**F**) Monocytes. Letters indicate significant differences between the bio-groups (*p* ≤ 0.05).

Significant differences were verified in total WBC counts, hematocrit, and in the total count of neutrophils, monocytes, lymphocytes, and thrombocytes. Regarding both WBC counts, hematocrit, lymphocytes, and thrombocyte peripheral numbers, a significant and sustained decrease was observed in all infected groups compared to fish sampled before infection, except for those sampled 3 h post-infection concerning the hematocrit value (Figure 1A–D). In the case of neutrophil numbers, a marked decrease was similarly evidenced, being significantly lower in the infected groups sampled 6, 24, and 48 h post-infection, compared to the non-infected group (Figure 1E). Monocyte counts were also lower in infected fish sampled at 9 and 48 h post-infection relative to numbers observed in the non-infected group (Figure 1F). On the other hand, the measurement of the other blood parameters (RBC, hemoglobin, MCV, MCH, and MCHC) did not show significant differences among the experimental groups. The complete set of results is available in Table S1.

### 2.2. Innate Humoral and Oxidative Stress Parameters

**Figure 2:**
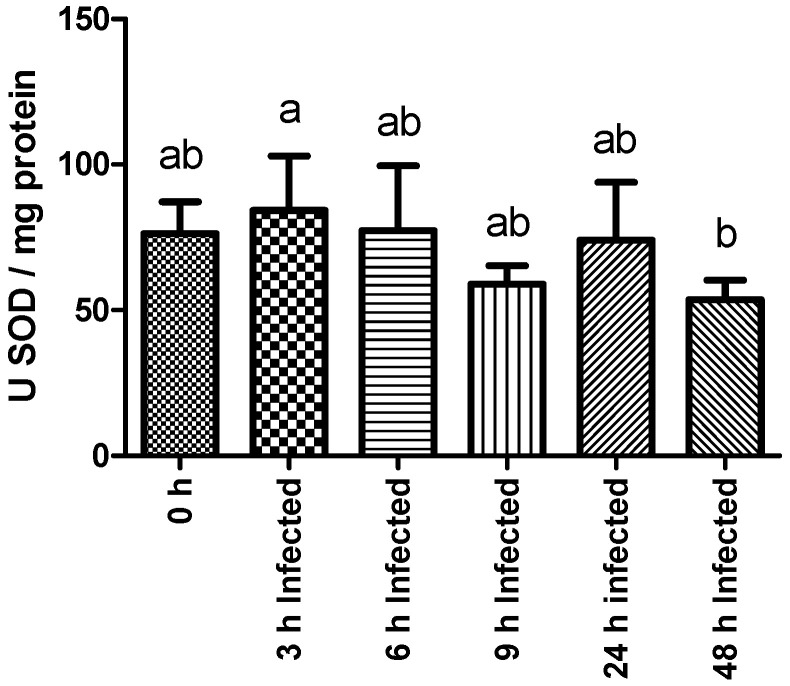
Liver SOD (superoxide dismutase) activity of rainbow trout sampled before (0 h) or 3, 6, 9, 24, and 48 h post-intraperitoneal infection with *Y. ruckeri* (means ± SD, *n* = 6). Letters indicate significant differences between the bio-groups (*p* ≤ 0.05).

Superoxide dismutase activity decreased significantly in infected fish sampled at 48 h compared to their counterparts sampled 3 h post-infection (Figure 2). No other statistically significant differences were found among experimental groups regarding plasma NO content and LYZ, GST, LPO, AP, and PER activities. The complete set of results is available in Table S2.

### 2.3. Immune Related Genes Analyzed by Real-Time qPCR

**Figure 3:**
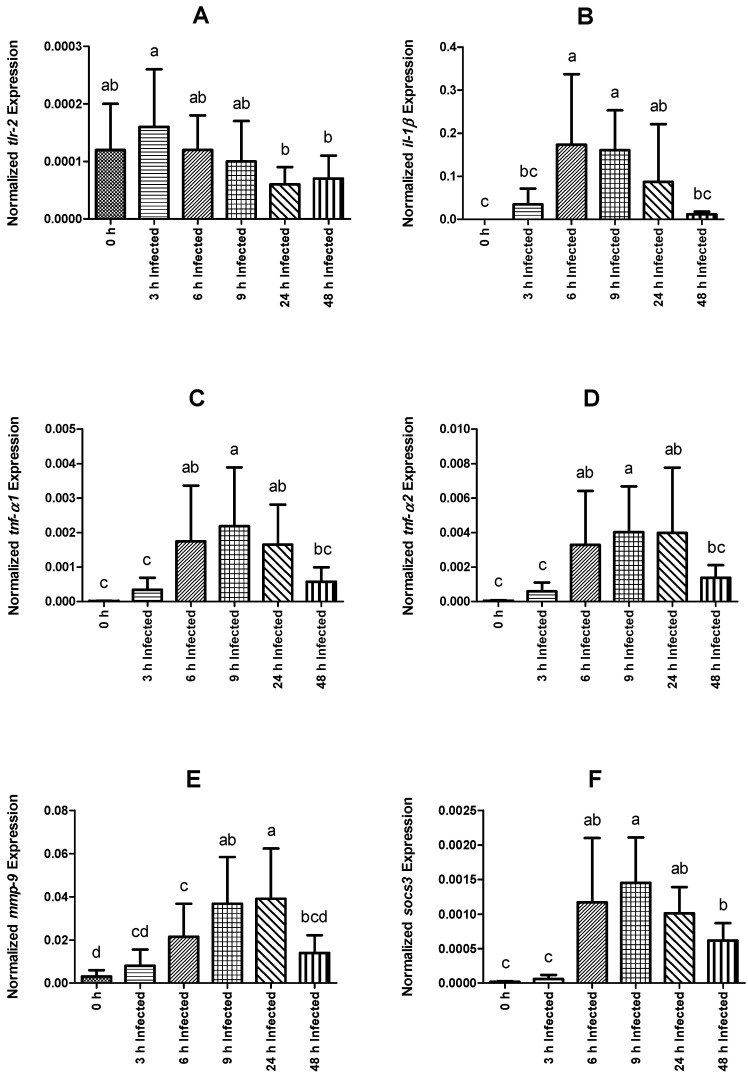
Normalized gene expression (ΔΔCq) of rainbow trout sampled before (0 h) or 3, 6, 9, 24, and 48 h post-intraperitoneal infection with *Y. ruckeri* (means ± SD, *n* = 6). (**A**) *tlr-2* (toll-like receptor 2); (**B**) *il-1β* (interleukin 1 beta); (**C**) *tnf-α1* (tumor necrosis factor alpha 1); (**D**) *tnf-α2* (tumor necrosis factor alpha 2); (**E**) *mmp-9* (matrix metalloproteinase 9); (**F**) *socs-3* (suppressor of cytokine signaling 3). Letters denote significant differences between bio-groups. In all cases, a statistical threshold of *p* ≤ 0.05 was used. The reference gene *ef-1α* (elongation factor-1 alpha) was used for normalization.

The immune response was analyzed in the head kidney. The expression level of the *tlr-2* gene did not differ between the control non-infected group and infected fish, but it was lower in fish sampled at 24 and 48 h than those at 3 h post-infection (Figure 3A). In comparison to fish sampled before infection, *il-1β*, *tnf-α1, tnf-α2, mmp-9,* and *socs-3* expression shows a trend to reach higher levels in fish sampled on the time frame between 6 and 24 h post-infection (Figure 3B–F). Additionally, regarding *socs-3* transcripts, these were still significantly higher at 48 h post-infection compared to the non-infected group. Among intraperitoneally injected fish, *il-1β*, *tnf-α1*, and *tnf-α2* expression increased over time, peaking at 6 h and decreasing back to basal levels at 48 h post-infection (Figure 3B–D). Infected fish showed an increase in the expression level of *mmp-9* over time, reaching maximum expression levels at 9 h post-infection, and returning basal expression at 48 h (Figure 3E). Similarly, *socs-3* was observed to peak at 9 h and gradually decreased to lower expression levels at 48 h post-infection (Figure 3F).

**Figure 4:**
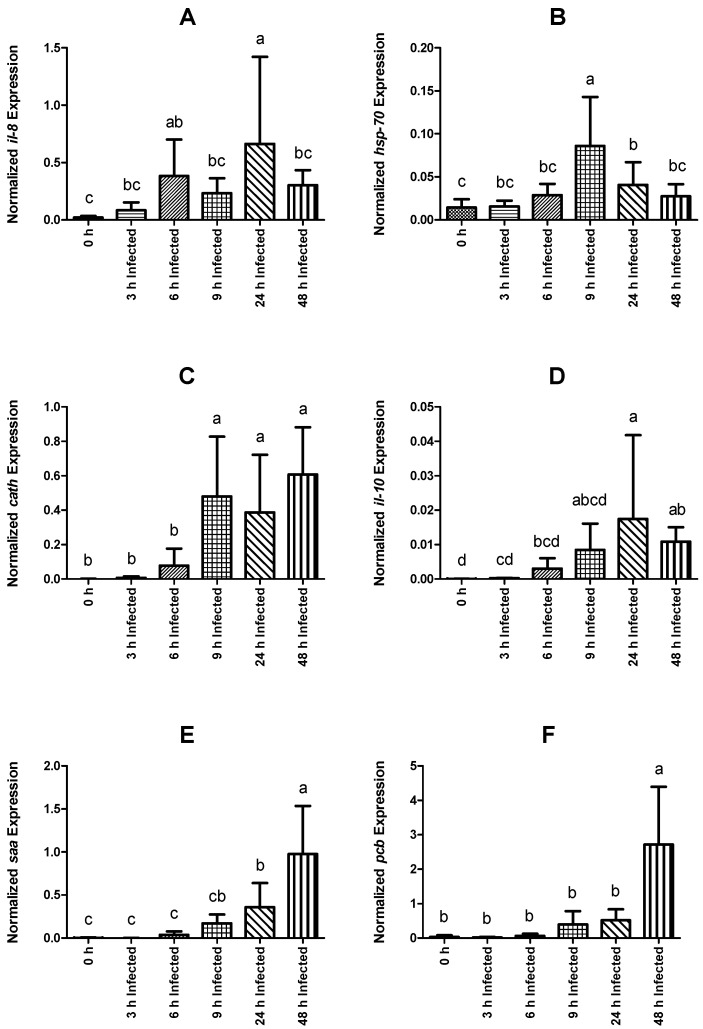
Normalized gene expression (ΔΔCq) of rainbow trout sampled before (0 h) or 3, 6, 9, 24, and 48 h post-intraperitoneal infection with *Y. ruckeri* (means ± SD, *n* = 6). (**A**) *il-8* (interleukin 8); (**B**) *hsp-70* (heat shock protein 70); (**C**) *cath* (cathelicidin); (**D**) *il-10* (interleukin 10); (**E**) *saa* (serum amyloid A); (**F**) *pcb* (precerebellin). Letters denote significant differences between bio-groups. In all cases, a statistical threshold of *p* ≤ 0.05 was used. The reference gene, *ef-1α* (elongation factor-1 alpha), was used for normalization.

With respect to *il-8*, expression in infected fish was higher at 6 and 24 h than that of non-injected fish (Figure 4A). Within infected groups, expression levels were higher at 24 h post-infection relative to all other sampling points. Nevertheless, the higher standard deviation (SD) level of the measurement at that time made it difficult to confirm upregulation at this point (24 h). With respect to the non-injected group, fish sampled at 9 and 24 h presented higher *hsp-70* expression (Figure 4B). In the infected groups, it peaked at 9 h post-infection and decreased over time to basal levels at 48 h. The *cath* expression was higher in infected fish sampled at 9, 24, and 48 h than that observed in the control, non-infected group. *cath* was upregulated from 6 to 9 h post-infection and remained high throughout the remaining sampling points (Figure 4C). Additionally, the level of expression of *il-10* was higher in infected fish sampled at 24 and 48 h compared with the non-infected group, with the peak at 24 h (Figure 4D). Nonetheless, the SD level raises an issue to confirm upregulation at 24 h.

**Figure 5:**
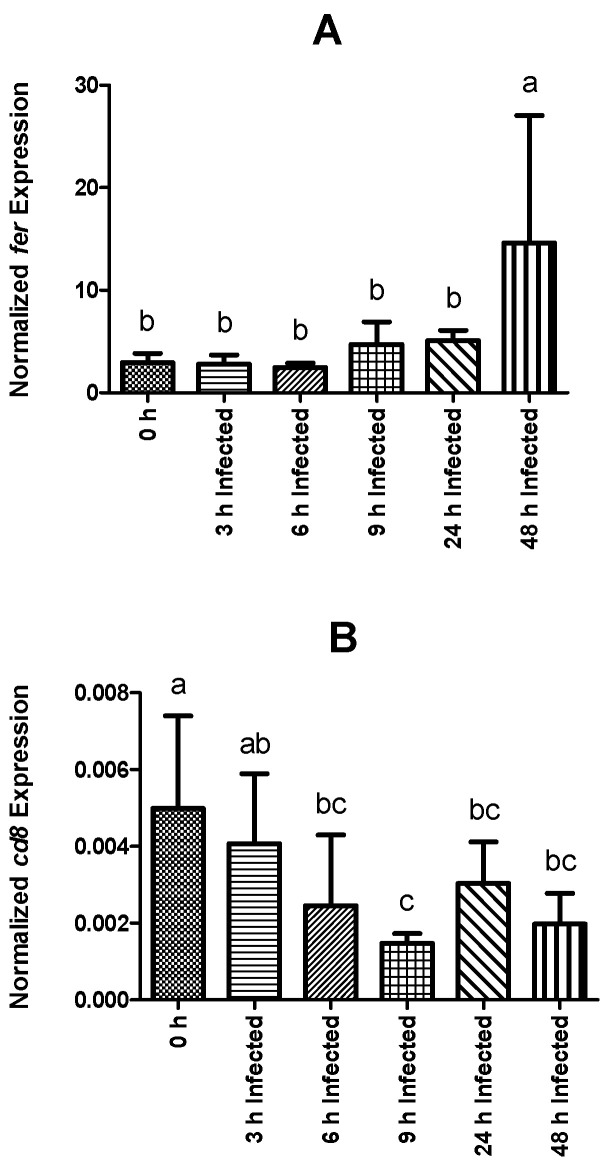
Normalized gene expression (ΔΔCq) of rainbow trout sampled before (0 h) or 3, 6, 9, 24, and 48 h post-intraperitoneal infection with *Y. ruckeri* (means ± SD, *n* = 6). (**A**) *fer* (ferritin); (**B**) *cd8* (T-cell surface glycoprotein CD8 alpha precursor). Letters denote significant differences between bio-groups. In all cases, a statistical threshold of *p* ≤ 0.05 was used. The reference gene *ef-1α* (elongation factor-1 alpha) was used for normalization.

**Figure 6:**
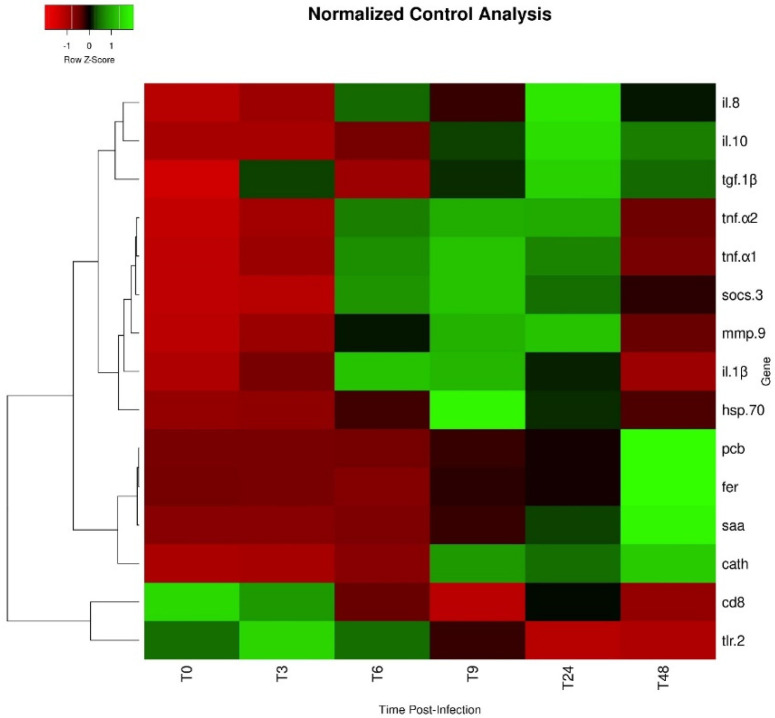
Heat map of gene expression in the head kidney of *O. mykiss* infected with *Y. ruckeri*. Lines represent genes and columns represent different time points. (T0: control uninfected; T3: 3 h post-infection; T6: 6 h post-infection; T9: 9 h post-infection; T24: 24 h post-infection; T48: 48 h post-infection). Different colors represent different levels of expression, ranging from low expression (red) to high expression (green).

Expression values of *saa* in infected fish were higher than levels observed in non-infected animals at 24 and 48 h, while *pcb* transcripts were only significantly higher at 48 h post-infection (Figure 4E and Figure 4F, respectively). Among fish subjected to the intraperitoneal injection, a time-dependent increase was observed regarding *saa* expression, which peaked at 48 h post-infection (Figure 4E). The expression level of *pcb* remained similar between infected groups sampled at 3, 6, 9, and 24 h post-infection, but significantly increased at 48 h (Figure 4F). Similar to *pcb, fer* gene expression was higher in fish sampled at 48 h post-infection than in non-infected fish and in all other infected groups (Figure 5A). Gene expression of *cd8* was observed to be downregulated over time, reaching the lowest levels at 9 h post-infection (Figure 5B). Moreover, infected fish sampled at 6, 9, 24, and 48 h showed significantly lower gene expression than those of the control group. On the other hand, *tgf-1β* did not show significant differences in its expression level between the different bio-groups, including the control group. The complete set of results is available in Table S3 and can be visualized through a heat map (Figure 6).

## 3. Discussion

The results of this study described some of the mechanisms engaged during the immune response of *O. mykiss* against infection with *Y. ruckeri*. The activation of defense mechanisms was verified at least in what the cellular response was concerned as the amount of WBC, neutrophils, monocytes, lymphocytes, and thrombocytes, which decreased in number in the blood as the infection developed, presumably as a result of the migration of these cells to the focus of the inflammatory response.

On the other hand, some negative effects were also shown on the mechanisms used to cope with the harmful effects of oxidative stress. The enzyme SOD catalyzes the dismutation of superoxide radicals and is considered part of the first line of defense against the formation of oxygen radicals that can be toxic for the organism [45]. Under normal physiological conditions, there is a continuous production of ROS that induces oxidative stress, resulting not only in the inactivation of enzymes, but also in the damage of genetic material and cell membranes, as well as other vital components [46]. An ongoing inflammatory process implies an increase in ROS production as a natural killing mechanism of innate immune cells, resulting in an exacerbation of oxidative stress and in higher activity of antioxidant enzymes [47]. Therefore, the observed decrease in the liver activity of SOD implies less regulation of ROS activity, thus resulting in an aggravated impact of ROS generation during the infection period. One could speculate about the presence of some mechanism deployed by *Y. ruckeri* to inhibit the expression of this enzyme. Indeed, it has been demonstrated that *Y. ruckeri* is able to compromise Myd88-mediated signaling as an evading strategy [48], thereby inhibiting pre-inflammatory signals.

In teleost fish, the kidney gathers excretory, immune, and endocrine tissues, but the anterior part, also known as the head kidney, is the main hematopoietic tissue of the fish, and the immune cells are produced and proliferating in response to immune stimuli. It is also a place where antigen-presenting cells interact with T-cells, triggering adaptive immune responses [49]; and it was precisely for this reason that the expression studies of genes related to the immune response were carried out.

Fish use TLR-like motifs in the process of recognition of antigens, triggering the expression of pro-inflammatory cytokines [50,51]. The *tlr-2* gene has been described in diverse fish species, including *O. mykiss* [52], as the protein that binds and identifies diverse microbial components, such as lipoproteins, lipoteichoic acid, and peptidoglycans [53]. In the present study, the lack of a more marked response in terms of a positive *tlr-2* regulation compared with uninfected control specimens would infer that, at least in the case of rainbow trout, TLR2 would not act as an important receptor of recognition of *Y. ruckeri*.

The regulation of the process of inflammation is the result of a complex mechanism of balance between pro- and anti-inflammatory cytokines, and it is a fundamental component of the immune response. IL-1β is one of the most studied pro-inflammatory cytokines in *O. mykiss* [54,55]. Bacterial ligands can stimulate macrophages inducing *il-1β* expression and activate lymphocyte proliferation [56]. Higher levels of expression of *il-1β* have been associated with infections with ectoparasites [57], virus [58], and exposure to bacterin derivate from *Y. ruckeri* [59]. Another typical pro-inflammatory cytokine, TNF-α, belongs to a superfamily of ligands and plays a key role as an important mediator of inflammation and immunity, which improves the phagocytosis and migration of leukocytes and also stimulates the expression of *il-1β* and *il-8* [60]. Currently, two *tnf-α* isoforms have been identified in *O. mykiss* [61,62]. In the present study, *il-1β* and both *tnf-α1* and *tnf-α2* expression levels peaked at 6 and 9 h post-injection, respectively, suggesting that these pro-inflammatory mediators would be responsible for initiating inflammatory reactions, triggering the innate immune response of *O. mykiss* against *Y. ruckeri* infection [24,63].

Additionally, *il-8*, belonging to the CXC chemokine subfamily, is known to have chemo-attractive properties on neutrophils in *O. mykiss* [64,65]. Expression levels of *il-8* in the head kidney of infected fish peaks at 24 h post-injection, suggesting that at this point, resident cells are actively communicating in order to recruit other immune cells, possibly for processes of antigen presentation. Previous reports have described increased levels of expression of *il-8* during *Y. ruckeri* challenge and bath vaccination, and also suggest that *il-8* is another key factor linked to the inflammatory reaction [66].

Interleukin 10 (*il-10*), *socs-3* and *tgf-β1*, belong to the group of regulatory factors. As a multifunctional cytokine, *il-10* has been reported in *O. mykiss* [67] with an immunosuppressive role, acting to minimize the possible deleterious effects to the host produced by an excessive response. Likewise, the expression of *socs* is controlled by cytokines and is dependent on the cell type. SOCS proteins are inhibitors of cytokine signaling pathways. Three SOCS isoforms (*socs-1, 2,* and *3*) have been reported in *O. mykiss* [68]. Additionally, TGF-β1 is a classical anti-inflammatory cytokine, with known roles in inducing immune cell differentiation into regulatory phenotypes [69], as well as in tissue regeneration [70,71,72]. Although no effects were observed on *tgf-β1* expression levels, both *il-10* and *socs-3* were modulated by infection. Regarding *il-10* expression values in the present experiment, its upregulation at 24 h post-injection might be associated with immunosuppressive functions, such as blocking chemokine receptors (*il-8*), reducing not only the effect of pro-inflammatory cytokines (*il-β*) [73] in a second phase of regulation but also the activation of macrophages/monocytes. These results also agree with the WBC and monocyte counts, which present their lowest levels at 24 and 48 h, respectively, and also with the gradual decrease in the expression levels of *il-1β* and *il-8*. Previous studies reported higher levels of expression of *il-10* that lasted for 3 days’ post-infection [24,74]. Furthermore, no pro-inflammatory cytokines were found to be upregulated after the higher levels of expression of *il-10*. Collectively, these data support the notion that *il-10* also functions as an anti-inflammatory factor. In contrast, the upregulation of the *socs-3* gene in the present experiment peaked earlier, at 9 h post-injection. These data would be related to an initial phase of modulation of the inflammatory response whereupon *il-1β* role is preponderant, with the aim of avoiding the deleterious effects that an excessive response could cause.

An increase in the expression levels of the *mmp-9* was evidenced with a peak at 24 h post-infection. MMP-9 is a matrix-degrading enzyme related to several biological processes, including inflammation, and it is highly expressed in migrating cells, such as neutrophils and macrophages [75]. MMP-9 has a key function in the process of controlling tissue contraction of collagen mediated by fibroblasts [76]. Thus, *mmp-9* plays a key function related to tissue remodeling and, in this case, its level of upregulation, would be linked to a mechanism to control the effects of the inflammatory response.

Cathelicidins are a family of proteins in which antimicrobial peptides (AMP) are included [77]. AMPs are gene-encoded peptides that eliminate bacteria by disrupting their cell membranes [78]. The observed upregulation of *cath* reveals the activation of this line of defense represented by the synthesis of AMPs. Previous studies have identified two *cath* genes in *O. mykiss*. According to Chang et al. [78,79], both CATH-1 and 2 peptides showed strong antibacterial activity.

Acute phase proteins are key components of the innate immune response against pathogens [80]. Those proteins are produced by the hepatocytes, and their synthesis is controlled by the macrophage activating factor, and a combination of cytokines, such as *tnf-α* and *il-1β* [81]. Among this group are the heat-shock proteins. In this experiment, the expression of *hsp-70* was only significantly upregulated at 9 h post-infection, the highest expression values within the evaluated timeframe, in line with the highest *tnf-α* and *il-1β* expression levels, too. These proteins are capable of providing protection to the cells by preventing aggregation or improper folding of other proteins [82]. Precerebellin (*pcb*) is another acute phase protein found in *O. mykiss* [83], and gene expression was observed to increase in infected rainbow trout [84]. This protein is the precursor of the brain-specific hexadecapeptide Cerebellin from chicken to humans [85] and encodes a final C1q domain, which is one of the first components of the complement cascade [86]. Its transcription was induced in fish liver following inflammatory stimulus [80] and released into the plasma [83], but its exact action mechanisms are still not clear. Regarding *pcb*, the present results agree with those reported by Raida and Buchmann [84], where this acute phase protein was upregulated in rainbow trout 3 days post i.p. injection with *Y. ruckeri*. In the present study, *pcb* was upregulated upon infection, but only 48 h post-injection, suggesting a role in the acute phase of infection. Importantly, the levels of expression of *cath*, *pcb, saa,* and *fer* were significantly upregulated at 48 h post-infection.

The homeostatic regulation of the iron concentration is part of the innate antimicrobial immune response [76]. Ferritin (*fer*) is an acute phase, iron-binding and trafficking protein and is considered an antimicrobial effector of the innate immune system due to its ability to reduce iron availability for microbes. Nevertheless, it is known that *Y. ruckeri* is able to grow under iron-limiting conditions due to the production of outer membrane proteins (Omps) [87,88].

In *O. mykiss*, *Y. ruckeri* infection-induced liver *il-1β* expression was positively correlated with that of *saa* [84]. The present results revealed a delay of this acute phase protein with respect to *il-1β,* which is upregulated at 24 h. Nevertheless, and being that these acute phase proteins are primarily produced in the liver, it may be that head kidney gene expression responsiveness happens in different timeframes from those in the liver.

Previous studies about the development of adaptive immunity in *O. mykiss* showed that the immune response is triggered by cytokines that stimulate lymphocytes to develop an adaptive immune response, leading to a more robust protective immunity [24]. CD8+ T cells are involved in the recognition of antigens that are presented as peptides on the cell surface [89]. Previous studies showed that there was no difference in the levels of expression of *cd8* between control and infected fish during a primary infection with *Y. ruckeri* [24]. Those findings contrast with our results, as the expression level of *cd8* decreased consistently compared with the uninfected group. This behavior could be explained by the migration of lymphocyte populations from the head kidney to the thymus for maturation.

In summary, results from this study showed for the first time short-term inflammatory and immune responses of rainbow trout following infection with *Y. ruckeri*. These well-orchestrated host responses were studied in a time-course fashion and thus can contribute to the establishment of biomarkers that may be used for the early detection of ERM. Importantly, the behavior of several genes in some time points (*il-1β* 6 h Figure 3B, *il-8* 24 h Figure 4A, *cath* 9 and 24 h Figure 4C, *il-10* 24 h Figure 4D, and *fer* 48 h Figure 5A) has to be taken as a trend due to the elevated level of the SD measured in the immune response of these genes. The levels of expression of innate immune genes related to bacterial invasion and elimination exhibited differences between individuals, which represents a possible explanation for the natural variation in susceptibility to infectious diseases. This could be helpful in the process of identification of immune markers that reflect a particular susceptibility status and also allow better comprehension of the immune response against this important pathogen.

## 4. Materials and Methods

### 4.1. Experimental Design

*O. mykiss* juveniles were transferred from a local fish farm (Castro Daire, Portugal) to CIIMAR facilities (Matosinhos, Portugal). The fish were fed 2 times a day (2% of the body weight) and quarantined for a period of 30 days. After this period, 72 fish (16.7 ± 4.4 g) were individually weighed and randomly distributed into 6 tanks (100 L) of a recirculating freshwater system (*n* = 12, animal initial density = 2 Kg/m^3^, photoperiod 12 h light/12 h dark). Physicochemical parameters, such as oxygen saturation (6.11 ± 0.47 mg/L), salinity (0.04 ± 0.02), and pH (8.22 ± 0.09), were monitored daily. Both temperature and ammonium/nitrite levels were kept constant throughout the trial (T = 18 ± 1 °C; NH_4_ and NO_2_ respectively under 0.50 and 1.22 mg/L).

### 4.2. Bacterial Growth and Inoculum Preparation

*Y. ruckeri* (QSP57.1) serovar I, isolated from *O. mykiss* in Portugal by Professor Alicia Toranzo (University of Santiago de Compostela, Santiago de Compostela, Spain), was cultured into tryptic soy agar (TSA, 1.5% of NaCl) (Pronadisa) plates for 24 h and isolated colonies were transferred to Erlenmeyer flasks containing 50 mL of tryptic soy broth (TSB, 1.5% of NaCl) (Difco). Bacteria were then cultured under continuous agitation (25 °C) for 18 h, and the concentration was read at 600 nm and finally adjusted to 2 × 10^8^ CFU/mL. The concentration was confirmed by plating the resulting cultures on TSA plates and counting CFU.

### 4.3. Bacterial Challenge

After 2 weeks of acclimatization to the recirculating water system, 50% of the individuals (3 tanks with 10 individuals each) were infected through peritoneal injection with 100 µL of the above suspension (2 × 10^7^ CFU/fish), while the other 50% were injected with the same volume of PBS and were thereby kept as a control group.

### 4.4. Sampling

Both infected and control groups were sampled immediately before infection (time 0), and then at 3, 6, 9, 24, and 48 h after challenge. Two fish per tank were randomly sampled at each time point (*n* = 6 for treatment) and euthanized in a solution of water with 2-phenoxyethanol (Sigma-Aldrich, St. Louis, MO, USA) (0.5 mL/L). Blood samples were collected from the caudal vessels using 1 mL syringes (previously prepared with 3000 U/mL of sodium heparin). Blood samples were then placed in 1.5 mL heparinized tubes and gently homogenized for hematological analysis, as described below. The remaining blood was centrifuged for 10 min at 10,000 g at 4 °C, and afterwards, plasma was collected and stored at −80 °C. The head kidney and liver were also aseptically collected for gene expression and oxidative stress analysis, respectively. After collection, the head kidney was stored in RNA later (with a proportion of 1/10 *w*/*v*) at 4 °C for the first 24 h and then stored at −80 °C. The liver was immediately frozen and stored at −80 °C until the oxidative stress analyses.

### 4.5. Hematological Analyses and Blood Smears

Before centrifugation of homogenized blood, a small aliquot was reaped for white blood cells (WBC) and red blood cells (RBC) counts, hematocrit (Ht), and hemoglobin determination (Hb, SPINREACT kit, ref. 1001230, Girona, Spain). Mean corpuscular volume (MCV), mean corpuscular hemoglobin (MCH), and mean corpuscular hemoglobin concentration (MHCH) were also calculated:

- MCV (µm^3^) = (Ht/RBC) × 10
- MCH (pg/cell) = (Hb/RBC) × 10
- MHCH (g/100 mL) = (Hb/Ht) × 100

The smears from heparinized blood were run through a single blood droplet and air dried. Afterwards, the slides were fixed with a solution of formaldehyde (Merck, Kenilworth, NJ, USA)-ethanol (Proclinica) (90% absolute ethanol to 10% of 37% formaldehyde) for 1 min [90]. Neutrophils were then marked by the detection of peroxidase activity, following a protocol described by Afonso et al. [91]. Subsequently, slides were stained with Wright’s stain (Haemacolor, Merck) and observed under oil immersion (1000×). Neutrophils, monocytes, lymphocytes, and thrombocytes were identified and differentially counted in a total of 200 cells/smear. Relative counts were further converted for absolute values (×10^4^/mL) of each cell type using WBC results.

### 4.6. Innate Humoral Immune Parameters

#### 4.6.1. Peroxidase (PER)

Total PER activity in plasma was measured following the procedure described by Quade and Roth [92]. To do so, 15 µL of plasma in duplicate was diluted in 135 µL of HBSS (Biowest, Riverside, MO, USA) without Ca^2+^ and Mg^2+^ in flat bottomed 96-well plates (Sarstendt, Budapest, Hungary). Then, 50 µL of 20 mM 3,3′,5,5′-tetramethybenzidine hydrochloride (TMB; Sigma-Aldrich) and 50 µL of 5 mM hydrogen peroxide (Sigma-Aldrich) were added, resulting in a change of color of the mixture that turned blue. The color change reaction was stopped after 2 min by adding 50 µL of 2M sulfuric acid (Sigma-Aldrich), and the optical density (OD) was read at 450 nm in a Synergy HT microplate reader (Biotek, Winooski, VT, USA). Two wells containing 150 µL of HBSS instead of plasma were used as blanks. PER activity (U/mL plasma) was determined by defining 1 U of PER as that which produces an absorbance change of 1 OD.

#### 4.6.2. Anti-Protease (AP) 

To do this, the method described by Ellis [93] was modified and adapted for 96-well microplates [94]. First, 10 µL of plasma was incubated with the same volume of trypsin (Sigma-Aldrich) solution (5 mg/mL in NaHCO_3_ 5 mg/mL, pH 8.3) for 10 min at 22 °C in polystyrene microtubes. Afterwards, 100 µL of phosphate buffer (NaH_2_PO_4_, 13.9 mg/mL, pH 7.0) and 125 µL of azocasein (Sigma-Aldrich) (20 mg/mL in NaHCO_3_, 5 mg/mL, pH 8.3) were added, and the mixture was incubated for 1 h at 22 °C. To stop trypsin activity, 250 µL of TCA were then added, and the microtubes were incubated for 30 min at 22 °C. Finally, the mixture was centrifuged at 10,000 g for 5 min at room temperature, and 100 µL of each supernatant was transferred to a 96-well plate in duplicate containing 100 µL of 1N NaOH. Phosphate buffer saline only was used as a blank solution in the protocol, and a reference sample was obtained using phosphate buffered saline instead of plasma. The percentage of trypsin activity was calculated as follows:

- % non-inhibited trypsin = (Sample absorbance × 100)/Reference sample
- % inhibited trypsin = 100 − % non-inhibited trypsin

#### 4.6.3. Lysozyme (LYS)

A turbidimetric assay was used to evaluate lysozyme activity following the method described by Costas et al. [95], with some modifications. Briefly, a solution of *Micrococcus lysodeikticus* was prepared (0.25 mg/mL in 50 mM Na_2_HPO_4_ buffer, pH 6.2). Plasma samples (10 μL) were added in duplicate to 150 μL of the above suspension in a microplate. 160 µL of Na_2_HPO_4_ buffer and 160 µL of bacterial suspension were also added in duplicate to serve as controls. The reaction was carried out at 25 °C, and the absorbance (450 nm) was measured after 0.5 and 20 min. A standard curve was prepared based on serially diluted, lyophilized hen egg white lysozyme (Sigma-Aldrich) in Na_2_HPO_4_ buffer (50 mM Na_2_HPO_4_ buffer, pH 6.2). The amount of LYS in the sample was calculated using the formula of the standard curve.

#### 4.6.4. Nitric Oxide (NO)

As both nitrite and nitrate are derivatives of endogenously produced NO, they are indicative of the NO amount in plasma. Therefore, the indications of the commercial Nitrite/Nitrate kit, colorimetric method, photometric endpoint determination (Roche, Cat. No. 11 746 081 001, Basel, Switzerland) were followed according to the manufacturer’s instructions and adapted to a 96-well plate.

### 4.7. Oxidative Stress

#### 4.7.1. Liver Homogenization

Liver tissues were homogenized with 10 volumes of ultrapure water, using a pellet mixer. A 200 µL aliquot was separated into a microtube with 4 µL 2,6-Di-tert-butyl-4-methylphenol (BHT; Sigma-Aldrich) 4% with methanol for lipid peroxidation (LPO) evaluation. One volume of tissue homogenate was mixed with one volume of potassium phosphate buffer (0.2 M, pH 7.4) and centrifuged at 10,000× *g* (4 °C) for 20 min. The supernatants were stored at −80 °C [96].

#### 4.7.2. Protein Concentration

Total protein concentration of liver homogenates was measured using Pierce™ BCA Protein Assay kit (Thermo-Scientific, Waltham, MA, USA), with bovine serum albumin as a standard, according to the manufacturer’s instructions as described by Costas et al. [97].

#### 4.7.3. Lipid Peroxidation (LPO)

LPO activity was determined following the method described by Bird and Draper [98]. In brief, 100 μL of cold 100% trichloroacetic acid (TCA) was added to each sample and thoroughly mixed. Then, 1 mL of 0.73% 2-thiobarbituric acid, Tris-HCl (60 mM) and 0.1 mM DTPA (Sigma-Aldrich) (pH 7.4) solution was added to each sample and blanks (200 μL ultrapure water + 4 μL of BHT 4% in methanol + 100 μL of TCA + 1 mL of TBA). The mixtures were incubated for 1 h at 100 °C in a laboratory oven, and then centrifuged for 5 min at 15,000 g. Finally, 200 µL of supernatant was transferred to a microplate in triplicate. The absorbance was measured at 535 nm, and LPO was expressed as nmol of thiobarbituric acid reactive substances (TBARS) formed per g of wet tissue [96].

#### 4.7.4. Catalase (CAT) 

CAT activity was measured as described by Clairborne [99] and was adapted to a microplate [96]. It was based on the consumption of the substrate (H_2_O_2_), observed as the decrease in absorbance. Briefly, 10 µL of previously diluted sample (K-phosphate buffer 100 mM, pH 7.4; 0.7 mg/mL final protein concentration) was added in triplicate to a UV-suited microplate, along with 140 µL of potassium phosphate (50 mM, pH 7.0) and 150 µL of 30% H_2_O_2_. The absorbance was read at 240 nm for 2 min (1 read every 15 s), and catalase activity was expressed in U per mg of protein, using the H_2_O_2_ molar extinction coefficient at 240 nm (43.6 M/cm).

#### 4.7.5. Superoxide Dismutase (SOD)

SOD activity was monitored according to Almeida et al. [100], using the cytochrome C method, with xanthine/xanthine oxidase as the source of superoxide radicals. A reaction solution containing 50 mM potassium phosphate buffer with 1 mM Na-EDTA (Sigma-Aldrich) (pH 7.8), 0.7 mM xanthine (Sigma-Aldrich), 0.03 mM cytochrome C (Sigma-Aldrich), 0.1 mM Na-EDTA, and 0.03 U/mL xanthine oxidase (Sigma-Aldrich) was added to previously diluted samples (0.3 mg/mL total protein content) in triplicate wells of a microplate. The absorbance was read at 550 nm for 3 min in intervals of 20 s. Activity is reported in units of SOD per mg of protein. One unit of activity was defined as the amount of enzyme necessary to produce a 50% inhibition of the cytochrome C reduction rate.

#### 4.7.6. Glutathione-S-Transferase (GST)

GST activity was determined following the method of Habig et al. [101] adapted to a microplate by Frasco and Guilhermino [102]. Briefly, 50 μL of each sample (previously diluted in K-phosphate buffer, 0.1 M pH 7.4; 0.7 mg/mL final protein concentration) was added to triplicate wells of a microplate. Then, 250 μL of a reaction solution (0.2 M potassium phosphate buffer, pH 6.5), 10 mM reduced glutathione (GSH; Sigma-Aldrich), and 60 mM 1-chloro-2,4-dinitrobenzene (CDNB, Alfa Aesar, Haverhill, MA, USA) was added to each well. Absorbance was recorded at 340 nm for 5 min at 20 s intervals. GST activity was expressed as mU per mg of protein, using the molar extinction coefficient at 340 nm (9.6 × 106 M/cm) [96].

### 4.8. Gene Expression Analysis

The extraction of the head kidney’s RNA was performed with an NZY total RNA isolation kit (NZYTech, Lisbon, Portugal), following the manufacturer’s instructions. After extraction, RNA samples were quantified, and purity was assessed by spectrophotometry using DeNovix DS-11 FX. Samples varied in RNA quantity from 123.83 ng/µL to 1144.44 ng/µL and presented 260:280 ratios between 1.89 and 2.13, respectively. NZY first-strand cDNA synthesis kit (NZYTech) was used for cDNA synthesis, also allowing the standardization of cDNA concentration. Reverse transcription was then performed on a Veriti DX 96-well Thermal Cycler (Applied Biosystems, Waltham, MA, USA). Real-time quantitative PCR was carried out in a CFX384 Touch Real-Time PCR Detection System (Biorad, Hercules, CA, USA), using 4.4 μL of diluted cDNA (20 ng/µL) mixed with 5 μL of The NZYSpeedy qPCR Green Master Mix^®^ and 0.3 μL (10 μM) of each specific primer in a final volume of 10 μL. Fifteen target genes were selected and studied according to their influence on the immune response (Table 1). Primer efficiency was tested for each gene, with results varying between 121.62 and 83.74%. Cycling conditions were identical among different genes, varying only in the annealing temperature, consisting of 10 min at 95 °C for initial denaturation, 40 cycles of 2 steps (95 °C for 15 s, and primer annealing temperature for each different gene for 1 min), 1 min at 95 °C followed by 35 s at the annealing temperature and ending with 95 °C for 0.5 s. Each sample reaction was carried out in duplicate. Target gene expression was normalized using the *ef-1α* gene as housekeeping, and subsequently, the Pfaffl method [103] was used for gene expression calculations.

### 4.9. Statistical Analysis

Data are presented as the mean and standard deviation of each experimental group. Data were analyzed for normality and homogeneity of variance and Log transformed before statistical treatment when needed. Data were analyzed by one-way ANOVA followed by a post hoc Tukey HSD test between all combinations of different groups. The level of significance used was p ≤ 0.05 for all statistical tests. The study of gene expression was carried out using Bio-Rad CFX Maestro (Bio-Rad) software. Heat maps were generated through the Heatmapper web server using the previously described gene expression data, normalized to the control, and using the Average Linkage clustering method and Pearson distance measurement method [104].

## 5. Conclusions

Regarding the immune response at the cellular level, a decrease in the parameters WBC, neutrophils, monocytes, lymphocytes, and thrombocytes was corroborated, while few alterations to oxidative stress variables were observed, with only a slight decrease in SOD activity. At the transcriptional level, the expression of immune-related genes points to an increase of pro-inflammatory cytokines (il-1β, tnf-α1, tnf-α2), chemokines (il-8), anti-inflammatory cytokines (socs-3, il-10), regulatory factors (mmp-9), AMPs (cath), and acute phase proteins (hsp-70, fer, pcb, saa), in a succession of phases of modulation of the immune response of O. mykiss infected with Y. ruckeri.

## Figures and Tables

**Table 1:** Forward and reverse primers for real-time qPCR.

Gene	Acronym	Efficiency (%)	Annealing Temperature (°C)	Amplicon Length (bp)	Acession nº	Primer Sequence (5’-3’)
Interleukin 1 Beta	*il-1β*	102.52	58	62	AJ278242.2	F: CGTCACTGACTCTGAGAACAAGT R: TGGCGTGCAGCTCCATAG
Interleukin 10	*il-10*	100.36	58	119	NM_001245099.1	F: GGATTCTACACCACTTGAAGAGCC R: GTCGTTGTTGTTCTGTGTTCTGTTGT
Toll-Like Receptor 2	*tlr-2*	110.42	58	129	XM_036958363.1	F: GATCCAGAGCAACACTCTCAACAT R: CTCCAGACCATGAAGTTGACAAAC
Heat Shock Protein 70	*hsp-70*	83.74	60	135	AB062281.1	F: CTGCTGCTGCTGGATGTG R: GCTGGTTGTCGGAGTAAGTG
Suppressor of Cytokine Signaling 3	*socs-3*	121.62	58	228	AM74872Z3	F:CACAGAGAAACCGTTAAAAGGACTATCC R: AAGGGGCTGCTGCTCATGAC
Ferritin	*fer*	91.94	58	213	NM_001160521.1	F: ACTGGGTGACCAACCTCCG R: GGGCTACTGGCTTATAGGAACG
Tumor Necrosis Factor Alpha 1	*tnf-α1*	106.25	58	208	NM_001124374.1	F: CAAGAGTTTGAACCTCATTCAG R: GCTGCTGCCGCACATAAAG
T-Cell Surface Glycoprotein CD8 Alpha Precursor	*cd8*	105.22	58	202	AF178054.1	F: CGACGACTACACCAATGACC R: TGTGGGCATCTTTTTGTTCTT
Matrix Metalloproteinase 9	*mmp-9*	98.27	58	212	NM_001124370.1	F: CATGGTGTCATTTGGAAAAGC R: CGAAGGAAAAAGGGAAGTGG
Transforming Growth Factor Beta 1-Like	*tgf-β1*	96.92	58	229	XM_021591332.1	F: AGCTCTCGGAAGAAACGACA R: CGGGGTTGTGGTGCTTATAC
Interleukin 8	*il-8*	94.14	58	120	XM_021625343.1	F: AGAGACACTGAGATCATTGCCAC R: CCCTCTTCATTTGTTGTTGGC
Serum Amyloid A	*saa*	111.73	58	157	XM_036980911.1	F: GACGCCAACTGGAAAAACTC R: CTGCTGAGTCCTCGTGTCCT
Precerebellin	*pcb*	91.60	58	111	XM_021611216.2	F: GAAACTGCCAACCCAAAATC R: CATGCTACTGCCACTCCAGA
Cathelicidin Isoform X1	*cath*	87.54	58	159	XM_036984508.1	F: CAAGAAGAGGCAAGGACAGC R: TGTTCGATGCAGGGAGAGTT
Tumor Necrosis Factor Alpha 2	*tnf-α2*	100.71	58	140	NM_001124374.1	F: AGAGGGGCCTTGAAAATAGCC R: TGCCGCACATAAAGCTGCTA
Elongation Factor-1 Alpha	*ef-1α*	86.32	55	159	NM_001124339.1	F: GGCAAGTCAACCACCACAG R: GATACCACGCTCCCTCTCAG

## Data Availability

Not applicable.

## Supplementary Materials

The following supporting information can be downloaded at: https://www.mdpi.com/article/10.3390/ijms23063096/s1.
