# Supplementary material for: Functional and Molecular Immune Response of Rainbow Trout (Oncorhynchus mykiss) Following Challenge with Yersinia ruckeri
Source: Int J Mol Sci. 2022 Mar 13;23(6):3096. doi: 10.3390/ijms23063096 (PMC8948951; doi:10.3390/ijms23063096)
Supplement: Supplementary file 1 [file ijms-23-03096-s001.zip › ijms-1619163-supplementary.pdf]

Table S1. Haematological parameters in *O. mykiss* i.p. injected with *Y. ruckeri* (INF) or placebo (PBS) and sampled at 3, 6, 9, 24 or 48 h post injection.

Parameters	0 h		3 h	6 h	9 h	24 h	48 h	*p value*
WBC	4.75 ± 1.27 a	PBS	4.61 ± 1.42 +	2.56 ± 0.52	2.68 ± 0.84	4.03 ± 1.09 +	4.31 ± 0.55 +	
		INF	2.30 ± 0.72 b	1.88 ± 0.30 b	1.06 ± 0.36 b	1.28 ± 0.60 b	0.61 ± 0.22 b	*< 0.001*
RBC	0.79 ± 0.15	PBS	0.80 ± 0.15	0.79 ± 0.07	0.92 ± 0.12 +	0.94 ± 0.07 +	1.00 ± 0.13 +	
		INF	0.79 ± 0.10	0.73 ± 0.14	0.66 ± 0.11	0.68 ± 0.14	0.58 ± 0.12	*< 0.001*
Haematocrit	23.33 ± 3.91 a	PBS	24.5 ± 5.46	26.50 ± 2.73	24.00 ± 6.29	27.00 ± 4.93 +	28.66 ± 1.50 +	
		INF	23.5 ± 2.88 a	19.00 ± 2.75 b	20.50 ± 2.94 b	17.5 ± 4.20 b	15.83 ± 3.18 b	*< 0.001*
Haemoglobin	2.81 ± 0.91	PBS	3.33 ± 0.67	3.32 ± 0.74	3.37 ± 0.83	3.08 ± 0.26	3.70 ± 0.41 +	
		INF	2.43 ± 0.71	2.83 ± 0.50	2.59 ± 0.28	3.03 ± 1.04	2.10 ± 0.65	*0.01*
MCV	287.20 ± 51.22	PBS	306.97 ± 66.71	337.46 ± 44.84	262.80 ± 72.05	284.59 ± 45.16	289.43 ± 41.83	
		INF	298.42 ± 31.64	268.00 ± 70.66	319.40 ± 94.13	282.27 ± 40.03	275.99 ± 47.81	*0.551*
MCH	35.77 ± 10.25	PBS	41.70 ± 6.81	42.49 ± 11.46	37.01 ± 8.78	32.76 ± 4.51	37.25 ± 5.21	
		INF	30.98 ± 8.78	38.82 ± 4.27	39.13 ± 3.22	38.62 ± 11.06	35.84 ± 6.92	*0.364*
MCHC	11.95 ± 2.97	PBS	13.95 ± 3.00	12.76 ± 3.39	13.12 ± 3.12	11.73 ± 2.17	12.92 ± 1.20	
		INF	10.50 ± 3.23	13.72 ± 2.54	12.96 ± 2.99	12.80 ± 3.66	13.12 ± 2.16	*0.690*
Neutrophils	0.10 ± 0.12 a	PBS	0.09 ± 0.09	0.06 ± 0.03 +	0.03 ± 0.03	0.05 ± 0.05	0.04 ± 0.04 +	
		INF	0.04 ± 0.03 ab	0.02 ± 0.01 bc	0.03 ± 0.03 abc	0.02 ± 0.01 bc	0.008 ± 0.004 c	*0.026*
Monocytes	0.12 ± 0.08 a	PBS	0.07 ± 0.07	0.12 ± 0.06 +	0.11 ± 0.11 +	0.09 ± 0.06	0.06 ± 0.03	
		INF	0.08 ± 0.07 ab	0.04 ± 0.02 ab	0.03 ± 0.02 b	0.05 ± 0.03 ab	0.02 ± 0.01 b	*0.014*
Lymphocytes	1.95 ± 0.58 a	PBS	1.72 ± 0.58	1.04 ± 0.34	1.18 ± 0.46	1.79 ± 0.51 +	2.05 ± 0.42 +	
		INF	0.90 ± 0.35 b	0.78 ± 0.18 b	0.62 ± 0.25 b	0.54 ± 0.39 b	0.20 ± 0.06 b	*< 0.001*
Thrombocytes	2.55 ± 0.72 a	PBS	2.73 ± 0.70 +	1.33 ± 0.28	1.36 ± 0.40 +	2.09 ± 0.75 +	2.14 ± 0.41 +	
		INF	1.25 ± 0.40 b	1.02 ± 0.29 b	0.38 ± 0.13 b	0.66 ± 0.29 b	0.37 ± 0.14 b	*< 0.001*

WBC (white blood cells, 10^4 / uL); RBC (red blood cells, 10^6 / uL); Haematocrit (%); Haemoglobin (g / dL); MCV (mean corpuscular volume, um^3); MCH (mean cell hemoglobin, pg / cell); MCHC (mean corpuscular hemoglobin concentration, g / 100 mL); Neutrophils (10^4 / uL); Monocytes (10^4 / uL); Lymphocytes (10^4 / uL); Thrombocytes (10^4 / uL). Values are presented as means ± SD (n = 6). If the difference was significant, according to one-way ANOVA ($p \leq 0.05$), a HDS Tukey *post hoc* test was used to identify differences in the experimental conditions. Letters represent differences among bio-groups. Symbol (+) indicate differences between PSB and INF groups.

Table S2. Oxidative and innate humoral parameters in *O. mykiss* i.p. injected with *Y. ruckeri* (INF) or placebo (PBS) and sampled at 3, 6, 9, 24 or 48 h post injection.

Parameters	0 h		3 h	6 h	9 h	24 h	48 h	*p value*
CAT	73.17 ± 8.74	PBS	78.65 ± 16.41	100.38 ± 6.79 +	77.44 ± 12.19	79.84 ± 14.72	62.35 ± 14.34	
		INF	83.73 ± 20.22	67.25 ± 13.02	56.82 ± 18.43	68.82 ± 16.24	58.25 ± 19.93	*< 0.001*
GST	145.18 ± 17.37	PBS	162,75 ± 20,06	158,84 ± 15,39	146,40 ± 17,39	160,42 ± 35,56	156,58 ± 30,41	
		INF	144.04 ± 32.94	123.76 ± 35.01	148.19 ± 8.84	169.39 ± 32.50	140.01 ± 12.97	*0.118*
LPO	29.63 ± 13.53	PBS	18,02 ± 4,86	23,47 ± 10,66	18,06 ± 19,10	18,79 ± 9,95	20,70 ± 8,35	
		INF	31.16 ± 13.51	15.53 ± 13.28	16.69 ± 4.54	17.89 ± 6.47	11.94 ± 4.39	*0.059*
SOD	76.30 ± 10.92 ab	PBS	69.23 ± 6.17	74.21 ± 21.53	80.43 ± 14.48	74.93 ± 22.58	68.59 ± 13.17	
		INF	84.35 ± 18.62 a	77.35 ± 22.17 ab	58.90 ± 6.35 ab	74.03 ± 19.92 ab	53.53 ± 6.79 b	*0.042*
NO	1.44 ± 0.31	PBS	1.05 ± 0.22	1.02 ± 0.14	1.18 ± 0.17	1.07 ± 0.21	1.25 ± 0.23	
		INF	1.28 ± 0.44	1.23 ± 0.51	1.15 ± 0.54	1.12 ± 0.20	1.10 ± 0.22	*0.398*
LYS	7.04 ± 3.48	PBS	6.98 ± 3.33	5.66 ± 4.04	4.79 ± 1.37	3.98 ± 2.89	3.21 ± 0.87	
		INF	4.85 ± 3.54	3.43 ± 1.86	3.04 ± 1.21	3.73 ± 2.05	6.45 ± 2.23	*0.083*
AP	80.66 ± 9.23	PBS	78.46 ± 6.82	79.81 ± 8.13	71.53 ± 13.46	75.62 ± 9.62	59.50 ± 28.60	
		INF	68.54 ± 15.50	79.96 ± 3.18	69.78 ± 18.67	63.05 ± 25.06	58.58 ± 32.39	*0.166*
PER	3.33 ± 2.80	PBS	4.65 ± 3.53	7.86 ± 7.06	5.06 ± 7.00	7.64 ± 4.60	14.05 ± 8.48	
		INF	3.42 ± 4.35	4.20 ± 5.98	8.25 ± 7.97	12.00 ± 6.00	5.25 ± 4.67	*0.200*

CAT (catalase, U / mg); GST (glutathione S-transferase, mU / mg); LPO (lipid peroxidation, TBARS, nmol / g wt); SOD (super-oxide dismutase, U / mg prot); NO (nitric oxide, conc. µM); LYS (lysozyme, µg / mL); AP (anti-protease, % I.T.); PER (peroxidase; U / mL). Values are presented as means ± SD (n = 6). If the difference was significant, according to one-way ANOVA ($p \leq 0.05$), a HDS Tukey *post hoc* test was used to identify differences in the experimental conditions. Letters represent differences among bio-groups. Symbol (+) indicate differences between PSB and INF groups.

Table S3. Immune genes expression in head kidney of *O. mykiss* i.p. injected with *Y. ruckeri* (INF) or placebo (PBS) and sampled at 3, 6, 9, 24 or 48 h post injection.

Parameters	0 h		3 h	6 h	9 h	24 h	48 h	*p value*
il-1β	0.00004 ± 0.00004 c	PBS	0.00021 ± 0.00025	0.00051 ± 0.00082 +	0.00039 ± 0.00058 +	0.00007 ± 0.00005	0.00014 ± 0.00008	
		INF	0.03441 ± 0.03710 bc	0.17305 ± 0.16413 a	0.16041 ± 0.09295 a	0.08679 ± 0.13465 ab	0.01188 ± 0.00590 bc	*< 0.001*
il-10	0.00005 ± 0.00006 d	PBS	0.00003 ± 0.00002	0.00012 ± 0.00008	0.00012 ± 0.00009	0.00002 ± 0.00001 +	0.00003 ± 0.00002 +	
		INF	0.00021 ± 0.00016 cd	0.00307 ± 0.00297 bcd	0.00846 ± 0.007 abcd	0.01744 ± 0.02438 a	0.01084 ± 0.00417 ab	*< 0.001*
tlr-2	0.00012 ± 0.00008 ab	PBS	0.00012 ± 0.00003	0.00012 ± 0.00006	0.00011 ± 0.00006	0.00011 ± 0.00007	0.00015 ± 0.00008	
		INF	0.00016 ± 0.00010 a	0.00012 ± 0.00006 ab	0.00010 ± 0.00007 ab	0.00006 ± 0.00003 b	0.00007 ± 0.00004 b	*0.008*
hsp-70	0.01420 ± 0.00970 c	PBS	0.01152 ± 0.00416	0.01165 ± 0.00583	0.01573 ± 0.00816 +	0.01112 ± 0.00267	0.00811 ± 0.00211	
		INF	0.01540 ± 0.00705 bc	0.02872 ± 0.01298 bc	0.08610 ± 0.05687 a	0.04054 ± 0.02649 b	0.02734 ± 0.01430 bc	*< 0.001*
socs-3	0.00002 ± 0.00001 c	PBS	0.00002 ± 0.00001	0.00012 ± 0.00004 +	0.00009 ± 0.00005 +	0.00001 ± 0.00000 +	0.00001 ± 0.00001 +	
		INF	0.00006 ± 0.00006 c	0.00117 ± 0.00093 ab	0.00145 ± 0.00066 a	0.00101 ± 0.00038 ab	0.00062 ± 0.00025 b	*< 0.001*
fer	2.93711 ± 0.86698 b	PBS	1.87830 ± 0.61184	1.82523 ± 0.47729	1.68590 ± 0.42815	2.81372 ± 0.66555	3.07931 ± 0.69135 +	
		INF	2.78107 ± 0.89083 b	2.45941 ± 0.43235 b	4.67731 ± 2.21677 b	5.05647 ± 1.00776 b	14.58315 ± 12.46717 a	*< 0.001*
tnf- α1	0.00001 ± 0.00001 c	PBS	0.00001 ± 0.00001	0.00004 ± 0.00006 +	0.00003 ± 0.00004 +	0.00002 ± 0.00002 +	0.00002 ± 0.00002	
		INF	0.00034 ± 0.00035 c	0.00175 ± 0.00161 ab	0.00218 ± 0.00171 a	0.00165 ± 0.00116 ab	0.00057 ± 0.00042 bc	*< 0.001*
cd8	0.00499 ± 0.00241 a	PBS	0.00434 ± 0.00327	0.00294 ± 0.00117	0.00147 ± 0.00042	0.00341 ± 0.00066	0.00345 ± 0.00090	
		INF	0.00407 ± 0.00183 ab	0.00246 ± 0.00184 bc	0.00148 ± 0.00025 c	0.00303 ± 0.00109 bc	0.00198 ± 0.00080 bc	*< 0.001*
mmp-9	0.00312 ± 0.00293 d	PBS	0.00289 ± 0.00164	0.00363 ± 0.00410 +	0.01245 ± 0.00893 +	0.01937 ± 0.00858 +	0.00408 ± 0.00252	
		INF	0.00808 ± 0.00740 cd	0.02144 ± 0.01532 c	0.03675 ± 0.02166 ab	0.03912 ± 0.02317 a	0.01398 ± 0.0082 bcd	*< 0.001*
tgf- β1	0.00703 ± 0.00364	PBS	0.00703 ± 0.00291	0.00698 ± 0.00275	0.00879 ± 0.00271	0.01137 ± 0.00497	0.00922 ± 0.00390	
		INF	0.00819 ± 0.00253	0.00740 ± 0.00190	0.00814 ± 0.00414	0.00901 ± 0.00482	0.00830 ± 0.00330	*0.08*
il-8	0.02055 ± 0.01206 c	PBS	0.01488 ± 0.00547	0.01749 ± 0.00866 +	0.01993 ± 0.01138	0.00871 ± 0.00421 +	0.01684 ± 0.00937	
		INF	0.08381 ± 0.06803 bc	0.38335 ± 0.31737 ab	0.23176 ± 0.13160 bc	0.66287 ± 0.75893 a	0.30345 ± 0.1294 bc	*< 0.001*
saa	0.00199 ± 0.00572 c	PBS	0.00114 ± 0.00177	0.00329 ± 0.00388	0.00570 ± 0.01206	0.00313 ± 0.00268 +	0.00389 ± 0.00389 +	
		INF	0.00164 ± 0.00143 c	0.03648 ± 0.04066 c	0.17046 ± 0.10128 cb	0.35819 ± 0.28131 b	0.97831 ± 0.55710 a	*< 0.001*
pcb	0.03056 ± 0.05078 b	PBS	0.01216 ± 0.01177	0.00531 ± 0.00166	0.01635 ± 0.02554	0.01895 ± 0.01867	0.01645 ± 0.01685 +	
		INF	0.01703 ± 0.01689 b	0.06257 ± 0.06456 b	0.39006 ± 0.39196 b	0.51929 ± 0.31724 b	2.71289 ± 1.68154 a	*< 0.001*
cath	0.00080 ± 0.00130 b	PBS	0.00185 ± 0.00138	0.00717 ± 0.00698	0.01627 ± 0.02625 +	0.00818 ± 0.00631 +	0.00370 ± 0.00163 +	
		INF	0.00746 ± 0.00805 b	0.07885 ± 0.09844 b	0.47951 ± 0.34845 a	0.38726 ± 0.33469 a	0.60668 ± 0.27541 a	*< 0.001*
tnf-α2	0.00004 ± 0.00003 c	PBS	0.00002 ± 0.00001	0.00011 ± 0.00016 +	0.00009 ± 0.00013 +	0.00005 ± 0.00004 +	0.00006 ± 0.00005	
		INF	0.00059 ± 0.00051 c	0.00328 ± 0.00314 ab	0.00401 ± 0.00267 a	0.00398 ± 0.00378 ab	0.00138 ± 0.0007 bc	*< 0.001*

Normalized gene expression (ΔΔCq). Values are presented as means ± SD (n = 6). If the difference was significant, according to one-way ANOVA (*p ≤0.05*), a HDS Tukey *post hoc* test was used to identify differences in the experimental conditions. Letters represent differences among bio-groups. Symbol (+) indicate differences between PSB and INF groups. Reference gene *ef-1α*.
